# Editorial: Plant polyphenols: new chemical and pharmacological approaches

**DOI:** 10.3389/fphar.2026.1885848

**Published:** 2026-06-09

**Authors:** Fabio Boylan, Małgorzata Kozyra, Wirginia Kukula-Koch

**Affiliations:** 1 Trinity College Dublin, Dublin, Ireland; 2 Uniwersytet Medyczny w Lublinie, Lublin, Poland

**Keywords:** chemistry, pharmacology, polyphenols, therapeutic applications, translational science

Plant polyphenols remain at the forefront of pharmacological research, offering a compelling interface between traditional medicinal knowledge and modern scientific innovation (1). Their chemical diversity and biological activities continue to drive interest across disciplines, from natural product chemistry to clinical pharmacology (2). The Research Topic *“Plant Polyphenols: New Chemical and Pharmacological Approaches”* highlights recent advances and emerging directions toward mechanistic understanding and therapeutic application.

The strong response to this call underscores the global relevance of polyphenol research. Following a rigorous peer-review process, seven articles were selected. These contributions, encompassing systematic reviews, meta-analyses, and experimental studies, collectively reflect a field that is evolving from descriptive exploration toward evidence-based and translational science. A unifying theme across the issue is the integration of chemical characterization with pharmacological insight, alongside an increasing emphasis on methodological rigor.

Zou et al. present a systematic review and critical evaluation of asperosaponin VI, a plant-derived metabolite gaining attention for its pharmacological properties. Although not strictly a polyphenol, its inclusion broadens the perspective of this issue to encompass structurally diverse natural compounds with therapeutic relevance. The authors provide a detailed analysis of its chemical features, processing technologies, and biological activities, emphasizing how preparation methods can significantly influence bioactivity. This work highlights the importance of aligning phytochemical processes with pharmacological outcomes, an essential step toward standardisation and reproducibility.

Curcumin, one of the most extensively studied phytochemicals, is addressed in two contributions. Tarlan et al. conduct a systematic review on the protective effects of curcumin against chemical-induced toxicity in the male reproductive system. Their findings consolidate evidence supporting curcumin’s antioxidant and anti-inflammatory roles in mitigating reproductive damage. At the same time, the study draws attention to inconsistencies in experimental design, reinforcing the need for standardized protocols.

Dai et al. extend the discussion of curcumin into the neurological domain through a meta-analysis of epileptic rodent models. By quantitatively synthesizing available data, they demonstrate that curcumin alleviates oxidative stress and inflammation, contributing to neuroprotection. Importantly, this study moves beyond qualitative observations to provide a more robust assessment of efficacy and mechanisms. Together, these two articles illustrate both the promise of curcumin and the ongoing challenges related to its bioavailability and clinical translation.

Quercetin, another prominent polyphenol, is examined by Yang et al. in the context of depressive symptoms. Their systematic review and meta-analysis of preclinical studies reveal consistent antidepressant-like effects, linked to modulation of oxidative stress, inflammation, and neurotransmitter systems. This work contributes to a growing recognition of polyphenols as modulators of neuropsychiatric health and highlights their potential role within the broader framework of the gut–brain axis. However, as emphasized by the authors, the translation of these findings into clinical practice remains an important next step.

The neuroprotective potential of polyphenols is further explored by Jalouli et al. in a comprehensive review focusing on neuroinflammation and neurodegenerative diseases. This article provides an integrative overview of how natural antioxidant polyphenols interact with key molecular pathways involved in disease progression, including oxidative stress, mitochondrial dysfunction, and immune activation. By linking mechanistic insights with pathological processes, the authors underscore the value of polyphenols as multi-target agents in complex disorders.

In addition to well-characterized compounds, this Research Topic also highlights underexplored plant sources. Tan et al. investigate the leaves of *Plukenetia volubilis* as a potential source of anti-*Helicobacter pylori* agents. Their findings demonstrate the antimicrobial potential of plant-derived compounds against a pathogen of significant clinical importance. This study reinforces the relevance of bioprospecting and the continued need to explore diverse botanical resources, particularly those informed by traditional knowledge.

Finally, Martiniakova et al. examine sea buckthorn and its flavonoids—isorhamnetin, quercetin, and kaempferol—in relation to bone and breast tissue health. Their review highlights the capacity of these compounds to influence pathways associated with skeletal integrity and hormone-responsive tissues. The dual focus on bone and breast health provides a nuanced perspective on tissue-specific and systemic effects, suggesting potential applications in conditions such as osteoporosis and hormone-related disorders.

Taken together, the contributions in this Research Topic illustrate several important trends shaping the future of polyphenol research. First, there is a clear shift towards evidence synthesis, as reflected in the number of systematic reviews and meta-analyses. This trend indicates a maturation of the field and a commitment to critically evaluate existing data. Second, there is an increasing focus on mechanistic understanding, particularly in relation to oxidative stress, inflammation, and cellular signalling pathways. Such insights are essential for bridging the gap between preclinical findings and clinical application (3).

Third, the integration of chemical and pharmacological approaches is becoming more pronounced. Advances in analytical techniques and processing technologies are enabling more precise characterization of bioactive compounds and their transformations (4). This integration is crucial for addressing challenges related to standardisation and reproducibility. Fourth, the recognition of polyphenols as multi-target agents aligns with the growing appreciation of complex, multifactorial diseases that require holistic therapeutic strategies (5).

Despite these advances, several challenges persist. Issues such as low bioavailability, variability in experimental models, and limited clinical evidence continue to hinder translation. Addressing these limitations will require interdisciplinary collaboration, improved study design, and innovative delivery systems. Moreover, maintaining a balance between enthusiasm for natural products and rigorous scientific validation remains essential.

In conclusion, this Research Topic provides a timely and insightful overview of new chemical and pharmacological approaches to plant polyphenols. The selected articles not only highlight the therapeutic potential of these compounds but also emphasize the need for methodological rigor and translational focus. We hope that this collection will stimulate further research and contribute to the development of effective, evidence-based applications of plant-derived compounds in modern medicine.

